# Self-directed learning *versus* traditional instructor-led learning for education on a new anaesthesia workstation: a noninferiority, randomised, controlled trial

**DOI:** 10.1016/j.bja.2025.03.043

**Published:** 2025-05-22

**Authors:** Caterina Gutersohn, Sandra Schweingruber, Maximilian Haudenschild, Markus Huber, Robert Greif, Alexander Fuchs

**Affiliations:** 1 Department of Anaesthesiology and Pain Medicine, Inselspital, Bern University Hospital, Bern, Switzerland; 2 Faculty of Medicine, University of Bern, Bern, Switzerland; 3 Department of Surgical Science, University of Turin, Turin, Italy

**Keywords:** continuous professional development, interprofessional education, medical education, patient safety, self-directed learning, video learning

## Abstract

**Background:**

It is fundamental for patient safety that medical devices are correctly and competently applied. Shift work, especially in anaesthesia, poses challenges to provide timely comprehensive learning opportunities. Because personnel shortages encourage cuts to staff education, self-directed learning might alleviate these difficulties.

**Methods:**

We conducted a single-centre, noninferiority, randomised, controlled trial in a large university-based anaesthesia department. Anaesthesia nurses and physicians were randomly assigned 1:1 to self-directed learning including a learning video (intervention) or to an instructor-led workshop (control). Both groups attended a 1-h teaching session on a new anaesthesia workstation. Around 3 months later, participants from both groups were assessed on 12 competences at an examination station. The defined primary outcome was the difference in success rates between groups. We hypothesised that the success rate of self-directed learning would be noninferior to instructor-led learning by a noninferiority margin of *Δ*=10%.

**Results:**

Data from 222 participants (97 anaesthesia nurses, 125 physicians) were analysed. Participants were aged between 32 and 44 yr; 35.6% had <5 yr of professional experience. The success rate difference between both groups was −0.9% (90% confidence interval: −3.8%–1.7%), confirming the noninferiority of self-directed learning.

**Discussion:**

Creating an educational video for the self-directed acquisition of necessary knowledge and skills to handle an anaesthesia workstation requires initial investment, but reduces substantially instructor time, as learners can study independently according to their needs. Video-supported self-directed learning of the handling of an anaesthesia workstation was not inferior to traditional teacher-led instruction.

**Clinical trial registration:**

ClinicalTrials.gov(NCT05530382).

**Editor's key points**

• Patient safety requires that medical devices are correctly and competently applied, but staff shortages and working schedules pose challenges in providing comprehensive learning opportunities.
• This single-centre noninferiority trial in a large university-based anaesthesia department compared self-directed learning with a video with an instructor-led workshop for education on a new anaesthesia workstation.
• Video-supported self-directed learning for operating an anaesthesia workstation was noninferior to traditional teacher-led instruction.
• Self-directed learning with an integrated learning video requires an initial financial investment to create the video, but reduced the need for instructors over time, an important advantage in staffing constrained environments.

Medical devices are indispensable to modern healthcare systems.^1^ Development of anaesthesia workstations over the past few decades has been rapid and continues.^2^ Although the usability of modern anaesthesia ventilators is constantly improving, targeted training of naïve users is still necessary.^3^ This creates a continuing need to train users on new devices to ensure patient safety.^4^ At the same time, personnel shortages in the healthcare system are a recognised problem,^5^ one that also impacts education,^6^ as it is becoming increasingly difficult to provide continuous training for employees in order to maintain professional competence.^7^ The proper and safe handling of medical devices is critical for ensuring appropriate patient care and safety.^8^

Traditionally, instructor-led face-to-face workshops offering hands-on practice with didactic lectures have been used to train personnel in the use of medical devices, which is time-consuming and resource-intensive. Constrained working conditions and shift work, especially in anaesthesia, critical care, and emergency hospital services, pose challenges for educators and learners when it comes to holding and attending timely and comprehensive training opportunities. However, adult learners desire more self-directed, efficient, and effective training opportunities.^9^

Complex medical devices such as anaesthesia workstations^10^ require acquisition of physiological and medical knowledge, technical aspects of devices, and psychomotor skills. It has been argued that learning and retaining complex psychomotor skills are more effective with the use of supplemental videos than traditional didactic classroom teaching,^11^ especially for procedural processes in everyday contexts.^12^ The dual-coding learning theory^13^ describes how videos activate the human brain by simultaneously conveying visual and auditory information, making it easier to understand the information and better integrate it into long-term memory.^14^ High-quality instructional videos involving these different sensory channels enhance learning, when learners are not forced to divide their attention between incompatible sources of instructional material such as images, sound, and text.^15^ Professionally produced educational videos should use easy-to-understand language and sounds^15^ and be short (preferably <10 min), as learners' attention wanes over time.^16^ According to cognitive load theory and constructivism, the complexity of the content should be minimised, whereas unnecessary and distracting information should be avoided to enable new information to be linked with existing knowledge at a given time.^17^

We conducted a literature search in PubMed using the terms ‘*self-directed*’ AND ‘*medical device*’ OR ‘*anaesthesia workstation*’ from inception to October 26, 2022, without further restrictions. We found no publications addressing video-based self-directed education in medical device training. The goal of this study was to determine whether the scores on a practical assessment of anaesthesia staff were noninferior after they participated in a video-supported self-directed learning session, compared with traditional instructor-led teaching sessions aiming to provide instruction in handling a new anaesthesia workstation. We hypothesised that self-directed learning is noninferior to traditional instructor-led learning.

## Methods

This study was conducted as an investigator-initiated, single-centre, randomised, controlled trial with a noninferiority design. The Bern Cantonal Ethics Committee waived the need for ethical approval on July 25, 2021, following the Swiss Act for Human Research (BASEC Nr. Req-2021-00837). The study was conducted in line with the Helsinki Declaration.^18^ The trial was prospectively registered on ClinicalTrials.gov (NCT05530382) on September 7, 2022. This report follows the applicable CONSORT statement,^19^ and a detailed study protocol was previously published.^20^ The study was conducted at the Department of Anaesthesiology and Pain Medicine, Bern University Hospital (Bern, Switzerland).

### Participants

We included the clinical anaesthesia staff of the anaesthesia department (i.e. certified nursing experts in anaesthesia care, anaesthesia residents, registrars, and consultants) older than 18 yr. We excluded participants who had already trained on or had practical experience with the new anaesthesia workstation. All participants received detailed study information, enabling written informed consent before assessment. Participation in the study was voluntary, and no incentives were offered.

A statistician, who was not involved in the teaching and assessment process, generated the block-stratified randomisation. A study nurse not otherwise involved in the teaching process randomised the study participants into two groups based on the learning method on September 19, 2022. The teaching phase started on October 4, 2022. The assessment of the participants happened between December 1, 2022, and January 31, 2023. All data from the assessment station were stored in coded form in a departmental REDCap database (REDCap Consortium, Vanderbilt University, Nashville, TN, USA).

The detailed teaching intervention has been published.^20^ It is summarised here:

The self-directed learning with an integrated learning video (intervention group) contained three parts.

1. Theory was taught through the learning video.
2. The practical hands-on component involved a worksheet for solving practical exercises on the anaesthesia workstation.
3. Self-check with theoretical questions, hands-on application tasks, and an answer key were used as a self-administered competence check.

The teacher-led workshop (control group) likewise contained three parts.

1. The theoretical content taught by the teacher was identical to the content in the video, and the same case study was used.
2. The same practical hands-on tasks were proposed as in the self-directed learning group.
3. The same application questions were used in the self-directed learning station.

The didactic development of the teaching programmes and the description of the production of the learning video are described in the study protocol.^20^

### Interventions

Participants were recruited before the mandatory training sessions that formed part of the departmental implementation process of a new anaesthesia workstation (Atlan, Draeger, Lübeck, Germany). As a preliminary reading, all participants received a summary of the anaesthesia workstation manual and were informed about the allocation of the training groups and the training process.

The self-directed learning group could independently visit the learning station, which was accessible to learners 24/7 for 8 weeks. All instructors in the instructor-led training were certified nurse experts in anaesthesia care with a subspecialisation in education. The study team informed and briefed the instructors about the learning content, the procedure, and the precise lesson plan they had to follow to ensure standardisation. After participants completed training, they had to attend an obligatory assessment station 4–8 weeks after the initial training, for which they could book an appointment individually online. We included only the assessments of study participants.

### Outcomes

The primary endpoint was the rate of passed assessments in the assessment station. This station was organised like a single objective structured clinical examination station from the anaesthesia nursing school evaluation examinations.^20^ Participants' learning results were assessed in a 1:1 setting at the assessment station. The examiners for the assessment station consisted of three certified nurse experts in anaesthesia care trained and briefed as examiners to ensure standardisation of the testing. Assessors were blinded to the intervention.

Each study participant received a case vignette and had to configure and operate the anaesthesia workstation correctly in relation to a fictitious, ventilated, and anaesthetised patient. The test was based on the framework curriculum for certified nurse experts in anaesthesia care in Switzerland,^21^ and included the following three criteria: principle of function; safety aspects, handling, and operating of the anaesthesia workstation; and theory–practice transfer. Each criterion included four questions that assessed the competency to handle the anaesthesia workstation. That resulted in 12 oral questions that were assessed by using a 4-point rating scale (0=does not apply, 1=rather does not apply, 2=applies, 3=applies very well). To pass the assessment, a score >22 was required (60% of a total of 36 points).^20^

Secondary endpoints included (1) overall score achieved in the study assessment; (2) number of post-training questions (questions were recorded manually by the educators in the instructor-led learning group at the end of the training session, and in the self-directed learning group by each individual via phone calls, e-mails, and direct questions); (3) learning-form preference (self-directed learning or traditional instructor-led learning), which was asked at the end of the competency test; and (4) use of resources and costs (personnel, learning materials). In our setting, we could use departmental rooms rent-free.

### Sample size calculation

Assuming a significance level of *α*=0.05, a final-exam success rate (‘passed’) in both groups (i.e. traditional instructor-led learning and self-directed learning) of 90%, and a noninferiority margin of *Δ*=10%, a sample size of 224 participants was calculated as necessary to establish the noninferiority of self-directed learning in comparison with an integrated learning video group with a power of 80%. This sample size is similar to previous studies.^22^, ^23^, ^24^ Because of high levels of personnel fluctuation in the department, block randomisation was performed in two stages. First, all clinical staff members able to participate in the study (*n*=260) were randomised. Then, to ensure block-size consistency, new employees replaced previously randomised dropouts from the same profession to maintain an appropriate sample size. Study personnel were excluded from the study.

### Statistical analysis

Participant characteristics are displayed as descriptive statistics by counts and percentages for categorical variables, by mean (standard deviation [sd]) for normally distributed quantitative variables, and by median (interquartile range [IQR]) for skewed quantitative variables. Distribution of participant baseline characteristics in both groups was compared using standardised mean differences.

Noninferiority of the primary outcome was assessed both via the crude difference in success rates in both groups and via the Mantel–Haenszel method to account for stratified randomisation in terms of provider as sensitivity analysis. The Mantel–Haenszel analysis provided a separate crude group comparison of certified nurse experts in anaesthesia care and physicians. Given the significance level of *α*=0.05, a 90% two-sided confidence interval of the difference in success rates was compared with the noninferiority margin of *Δ*=10%.

As a secondary endpoint, group differences in total score were analysed using a generalised linear model (GLM) with a beta distribution to account for the lower and upper bounds of the total score, where the total score was transformed into the (0,1) interval. All other subgroup analyses with respect to the outcome score (e.g. by age, sex, profession, level of education, and experience in anaesthesia) were assessed as exploratory and based on standard statistical tests for categorical and numerical data. The results of the exploratory analysis of secondary endpoints are presented in the Supplementary material (Supplementary Table S1 and Supplementary Fig. S1).

The main analysis was performed as an intention-to-treat analysis. A sensitivity analysis was performed per protocol. If data were missing, no imputation methods were used, and the final analysis represents a complete case analysis. The statistical software used for all analyses was R version 4.0.2 (R Foundation for Statistical Computing, Vienna, Austria).^25^

## Results

**Fig 1:**
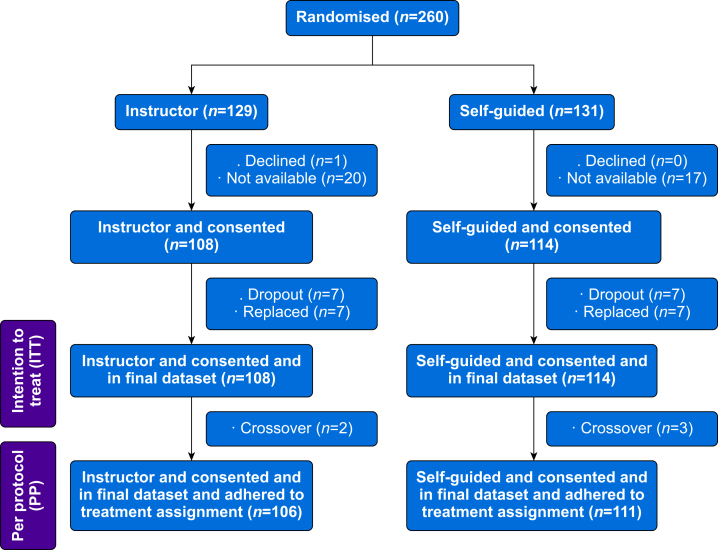
CONSORT flow diagram.

In total, 260 participants were randomised, 129 to the instructor-guided and 131 to the self-directed learning sessions. We excluded 38 participants (21 in the instructor-guided and 17 in the self-directed group) as informed consent was unavailable or declined. Finally, 222 participants (97 certified nurse experts in anaesthesia care and 125 physicians) were included in the final analysis, as displayed in the CONSORT diagram (Fig 1). There were seven dropouts in each group, all replaced, resulting in 108 participants in the instructor-guided group and 114 in the self-directed treatment group. For the per protocol analysis, five crossover participants were excluded (three in the instructor-guided and two in the self-directed group). Detailed participant baseline characteristics are given in Table 1.

**Table 1:** Baseline characteristics of the participants. Data are presented as *n* (%) or median (Q1; Q3 or SMD). SMD, standardised mean difference.

	Instructor-led	Self-directed	SMD
	(*n*=108)	(*n*=114)	
Age (yr)	37.0 (33.0; 44.2)	37.0 (32.0; 44.0)	0.03
Sex (female)	72 (66.7)	63 (55.3)	0.24
Profession			0.08
Physician	63 (58.3)	62 (54.4)	
Nurse	45 (41.7)	52 (45.6)	
Level of education			0.20
Resident	36 (33.3)	28 (24.6)	
Board-certified anaesthetist	27 (25.0)	34 (29.8)	
Certified expert in anaesthesia care	45 (41.7)	52 (45.6)	
Experience			0.17
0–5 yr	35 (32.4)	44 (38.6)	
6–10 yr	31 (28.7)	25 (21.9)	
>10 yr	42 (38.9)	45 (39.5)	

### Primary outcome

**Fig 2:**
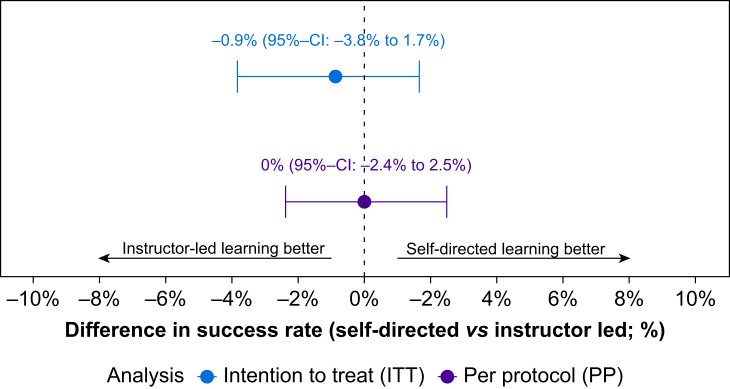
Noninferiority margin of the primary outcome difference in success rate. 95% CI, 95% confidence interval.

In the instructor-guided group, 100% of participants (*n*=108/108, 95% confidence interval [95% CI]: 96.6–100%) passed the test *vs* 99.1% of participants (*n*=113/114, 95% CI: 95.2–100%) in the self-directed group. The difference in the success rate between the instructor-guided and self-directed group was −0.9% (90% CI: −3.8%–1.7%), demonstrating noninferiority (Fig 2). Detailed analyses of the primary outcome are displayed in Table 2. The exploratory subgroup analysis showed a statistically significant decrease in the total score of −0.6 points (95% CI: −1.0 to −0.2 points, *P*=0.03) per decade of participant age (Supplementary Table S1 and Supplementary Fig. S1).

**Table 2:** Primary outcome according to an intention-to-treat (ITT) analysis. Data are presented as *n* (%) or median (Q1; Q3). 95% CI, 95% confidence interval. ∗The analysis of the total score with respect to the treatment group by means of a generalised linear model with a beta distribution resulted in a *P*-value of 0.78. **^†^**Per protocol analysis: 100% success rate in both groups (106/106 passed in instructor-led, 111/111 passed in self-directed). Modified ITT analysis of the primary outcome is identical to the ITT analysis. ^‡^The Cochran–Mantel–Haenszel test with respect to profession (physician and nurse) resulted in a *P*-value of 0.35. CIs of the weighted odds ratio could not be computed because of the low number of failed tests (e.g. only one participant failed).

	Instructor-led	Self-directed	*P*
	(*n*=108)	(*n*=114)	
Points			
Total points (maximum 36 points)	34.0 (32.0; 35.0)	34.0 (32.2; 35.0)	0.73*∗*
Principle of function (12 points)	11.0 (10.0; 12.0)	11.0 (10.0; 12.0)	0.26
Safety aspects (12 points)	12.0 (11.0; 12.0)	12.0 (11.0; 12.0)	0.20
Handling and operating/theory–practice transfer (12 points)	12.0 (11.0; 12.0)	12.0 (11.0; 12.0)	0.84
Outcome categories			
Primary endpoint^†^			>0.99^‡^
Failed	0 (0%, 95% CI: 0–3.4%)	1 (0.9%, 95% CI: 0–4.8%)	
Passed	108 (100%, 95% CI: 96.6–100%)	113 (99.1%, 95% CI: 95.2–100%)	
Detailed categories			0.97
A: Excellent (100%/36 points)	21 (19.4)	22 (19.3)	
B: Very good (90–99%/33–35 points)	59 (54.6)	63 (55.3)	
C: Good (80–89%/29–32 points)	21 (19.4)	23 (20.2)	
D: Satisfactory (70–79%/26–28 points)	5 (4.63)	3 (2.63)	
E: Sufficient (60–69%/22–25 points)	2 (1.85)	2 (1.75)	
F: Failed (<60%/<22 points)	0 (0)	1 (0.9)	

### Secondary outcomes

The overall scores for the outcome categories ‘failed’ to ‘excellent’ displayed no relevant differences between groups (Table 2). The number of post-training questions were significantly higher in the instructor-led group (*n*=713 questions) than in the self-directed group (*n*=2 questions). Teaching-method preferences differed significantly: 83.3% (*n*=90/108) in the instructor-guided treatment arm favoured traditional teacher-led workshop; 60.5% (*n*=69/114) of the self-directed workshop favoured the traditional teacher-led workshop (*P*<0.001). However, these preferences did not influence the primary outcome.

The costs for the video production were CHF 5382. The costs for the self-directed format were CHF 559 for additional teaching material. The costs for the teacher-led workshop were CHF 5905, which included the teaching material and the instruction and payment of the teachers. A detailed cost breakdown analysis is provided in Supplementary Figure S2.

## Discussion

This single-centre, noninferiority, randomised, controlled trial compared self-directed learning using a video accompanied by a hands-on simulation with a traditional teacher-led workshop on an anaesthesia workstation. We found no differences in success rates (primary outcome), confirming noninferiority. Both teaching methods achieved a high pass rate, which aimed to get the anaesthesia providers ready to use the new anaesthesia machine. However, the costs of each method differed substantially. Additionally, in both groups, we found a statistically significant decline in points achieved for each decade of participant age, and more senior participants preferred traditional teaching. That suggests besides the advantages of self-learning, in some cases, traditional teaching formats might be offered to satisfy learner needs, as no difference in the learning outcome was found.

Continuous professional development is essential to maintain the competencies needed to handle medical devices correctly, which are fundamental for patient safety. Traditional hands-on training in medical devices through teacher-led face-to-face workshops is time-consuming and resource-intensive. There is a shortage of healthcare workers in acute care disciplines, and limited personnel resources are a daily problem in most hospitals. This creates a dilemma between staffing for services and training employees, which is essential for learning and maintaining professional competences.

The employees of the Bern anaesthesia department already had experience with self-directed learning in medical device training (e.g. syringe pumps, monitor handling). However, no specific videos were used in those settings. Nonetheless, the study participants significantly preferred more traditional teacher-led workshops without videos. Interestingly, this subjective rating of teaching preference did not influence the learning outcome, showing the feasibility of the teaching concept.

It is well known that examinations and testing influence learning.^26^, ^27^, ^28^, ^29^ However, we cannot comment on the effect of our assessment station on the learning outcome as that was beyond the scope of the study. The competency test used for this study is based on an established and reliable test for anaesthesia nursing students.^20^

Some participants reported attending the self-study session with colleagues. This ‘learning together’ included self-organised learning^30^ and might have stimulated peer learning, as modern technology and social interactions in learning align well with social-cognitive learning theory, which claims that learning occurs in a social context through observation, imitation, and modelling.^31^

Teaching complex medical devices using a self-guided method could help optimise educational resources. With regard to cost, the reduced expenses for instructors in the self-guided learning station can compensate for the initial expenses of video production. Not having to organise teaching sessions for groups of employees but having an open space for individual learning whenever learners have time is an advantage of the self-learning approach and relieves the pressure on the administrative staff in charge of planning and scheduling. Also, videos can be watched wherever people are and whenever they want from their mobile devices.

Our study also demonstrated that the intervention was feasible in an interprofessional setting, without differences between professions, levels of education, or experience in anaesthesia. In Switzerland, interprofessional education is introduced early in the medical curriculum.^32^ In our study, both professions were equally well trained in using anaesthesia workstations.

According to Miller's Pyramid,^33^ our study showed that self-guided video learning combined with a practical assessment station can train behavioural competencies up to level three, as the learners needed to demonstrate their learned competencies in an objective exam before using the anaesthesia workstation on patients. Problem-solving behaviour is an important part of cognitive activation. It is highly relevant to the learner as it is crucial for safely applying the device to the patient. A mandatory competence assessment not only stimulates the learners to acquire competencies but also makes it possible to check if what is learned can be transferred into simulated practice.^34^^,^^35^

This study has several limitations. The single-centre design might hinder generalisability of the results. We had a high number of dropouts (around 20%) because of personnel changes (rotation of residents and nurses). Even though we did not achieve the calculated sample size, our data demonstrate the noninferiority of self-directed learning. Because of the different working schedules of the study participants, there was a gap of several weeks between training and testing for some participants, which might have influenced the results. However, because the assessment station was an exam on practical applied skills, participants could not simply learn the answers by heart, as with a multiple-choice test. Because of logistic reasons, only one assessor rated the study participants' performance. That precluded computation of interrater reliability. However, all assessors had long-lasting experience in medical device-related exams.

### Conclusions

Two 1-h learning formats (self-directed learning compared with teacher-led teaching) to acquire the needed skills to handle an anaesthesia workstation revealed no differences in success rates. Self-directed learning with an integrated learning video requires an initial financial investment to create the video, but reduces the need for instructors over time. Unlike traditional instructor-led teaching, this self-directed intervention allowed participants to schedule learning time independently, according to their needs. This might be an advantage for ensuring timely, continuous professional education in the acute care setting with ever-changing shift work. Even though participants had a self-reported preference for traditional, instructor-led learning, this did not influence the learning outcomes.

## Authors’ contributions

Conceptualisation: CG, RG.

Resources: CG, MHa, RG, AF.

Data curation: CG, SS, MHa, MHu.

Investigation: CG, SS, MHa.

Writing the original draft: CG, RG, AF.

Project administration: CG.

Methodology, formal analysis: MHu.

Supervision: RG, AF.

Reviewed and edited the manuscript: all authors.

Discussed the manuscript draft and contributed to the final version of the manuscript: all authors.

Agreed on the final version: all authors.

## Data sharing

The anonymised data set generated during the present study will be made available by the primary investigator upon reasonable request from researchers with suitable and answerable research questions and local ethics committee approval, in line with the Swiss Human Research Act.

## Funding

Department of Anaesthesiology and Pain Medicine, Inselspital, Bern University Hospital.

## Declaration of interest

The authors declare that they have no conflicts of interest.
