# Supplementary material for: Self-directed learning versus traditional instructor-led learning for education on a new anaesthesia workstation: a noninferiority, randomised, controlled trial
Source: Br J Anaesth. 2025 May 22;135(4):990–6. doi: 10.1016/j.bja.2025.03.043 (PMC12674044; doi:10.1016/j.bja.2025.03.043)
Supplement: Multimedia component 1 [file mmc1.docx]

**Legends to Supplementary Material**

**Supplementary Table 1.** Exploratory subgroup analyses for the total score for (A) sex, (B) profession, (C) level of education and (D) years of experience.

**Supplementary Figure 1.** Exploratory subgroup analyses of the instructor-guided (blue) and the self-guided (red) groups for (A) age, (B) sex, (C) profession, (D) level of education and (F) years of experience in anaesthesia.

**Supplementary Figure 2.** Waterfall plot of the cost calculation of training creation and training for (A) self-directed and (B) instructor led teaching.

| 1. **Sex** | **Female** | | | **Male** | | |
| --- | --- | --- | --- | --- | --- | --- |
|  | **Instructor-led** | **Self-directed** | ***p*** | **Instructor-led** | **Self-directed** | ***p*** |
|  | *N=72* | *N=63* |  | *N=36* | *N=51* |  |
| Total score | 34.0 [33.0;35.0] | 34.0 [32.5;35.0] | 0.79 | 34.0 [31.8;35.0] | 33.0 [32.5;34.0] | 0.84 |

| 1. **Profession** | **Nurse** | | | **Physician** | | |
| --- | --- | --- | --- | --- | --- | --- |
|  | **Instructor-led** | **Self-directed** | ***p*** | **Instructor-led** | **Self-directed** | ***p*** |
|  | *N=45* | *N=52* |  | *N=63* | *N=62* |  |
| Total score | 35.0 [33.0;36.0] | 34.0 [32.0;36.0] | 0.44 | 34.0 [32.0;35.0] | 34.0 [33.0;34.0] | 0.81 |

| 1. **Level of education** | **Certified expert in anaesthesia care** | | | **Board-certified anaesthetist** | | | **Resident** | | |
| --- | --- | --- | --- | --- | --- | --- | --- | --- | --- |
|  | **Instructor-led** | **Self-directed** | ***p*** | **Instructor-led** | **Self-directed** | ***p*** | **Instructor-led** | **Self-directed** | ***p*** |
|  | *N=45* | *N=52* |  | *N=36* | *N=28* |  | *N=27* | *N=34* |  |
| Total points | 35.0 [33.0;36.0] | 34.0 [32.0;36.0] | 0.44 | 33.5 [32.0;35.0] | 33.0 [32.8;34.0] | 0.99 | 34.0 [33.0;34.0] | 34.0 [33.0;34.0] | 0.73 |

| 1. **Experience** | **0-5 yrs** | | | **6-10 yrs** | | | **>10 yrs** | | |
| --- | --- | --- | --- | --- | --- | --- | --- | --- | --- |
|  | **Instructor-led** | **Self-directed** | ***p*** | **Instructor-led** | **Self-directed** | ***p*** | **Instructor-led** | **Self-directed** | ***p*** |
|  | *N=42* | *N=45* |  | *N=35* | *N=44* |  | *N=31* | *N=25* |  |
| Total points | 34.0 [32.0;35.0] | 34.0 [31.0;36.0] | 0.68 | 34.0 [33.0;35.0] | 34.0 [33.0;35.0] | 0.70 | 34.0 [32.5;35.0] | 33.0 [33.0;34.0] | 0.46 |

Data given in median [Q1;Q3]

**Supplementary Table 1.** Exploratory subgroup analyses for the total score for (A) sex, (B) profession, (C) level of education and (D) years of experience.

**
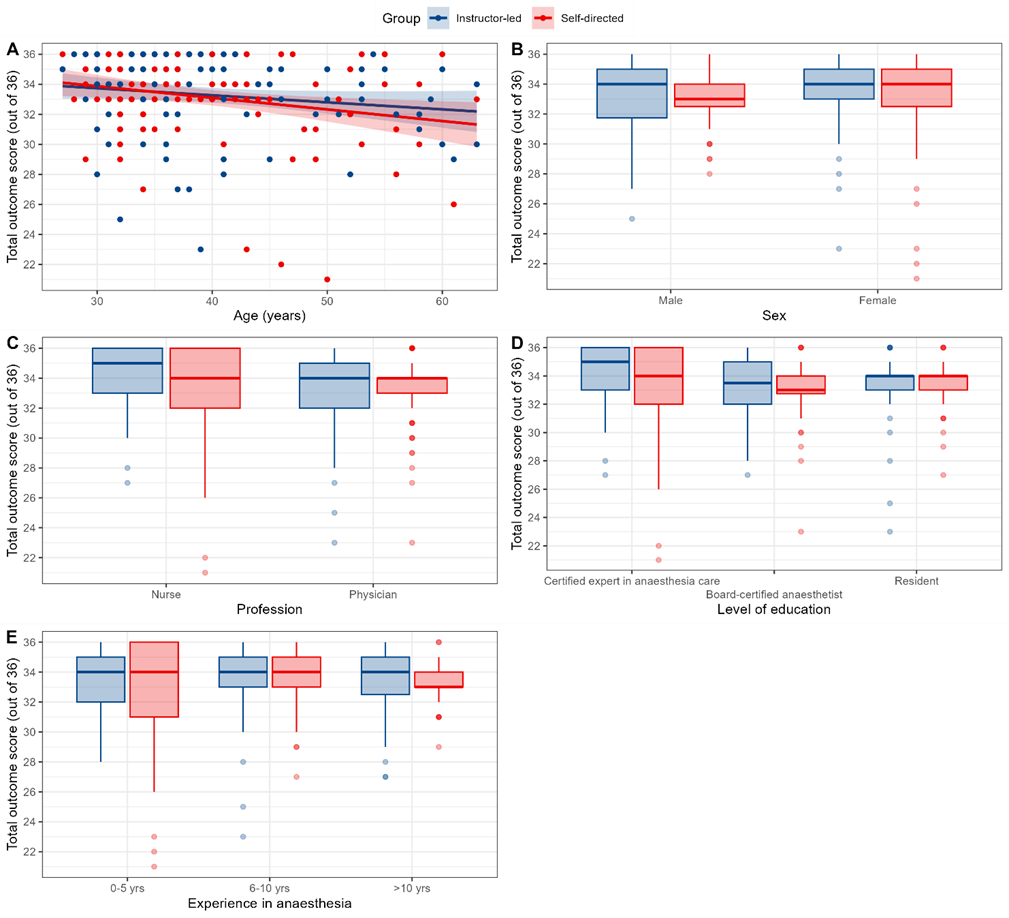
**

**Supplementary Figure 1.** Exploratory subgroup analyses of the instructor-guided (blue) and the self-guided (red) groups for (A) age, (B) sex, (C) profession, (D) level of education and (F) years of experience in anaesthesia.

**
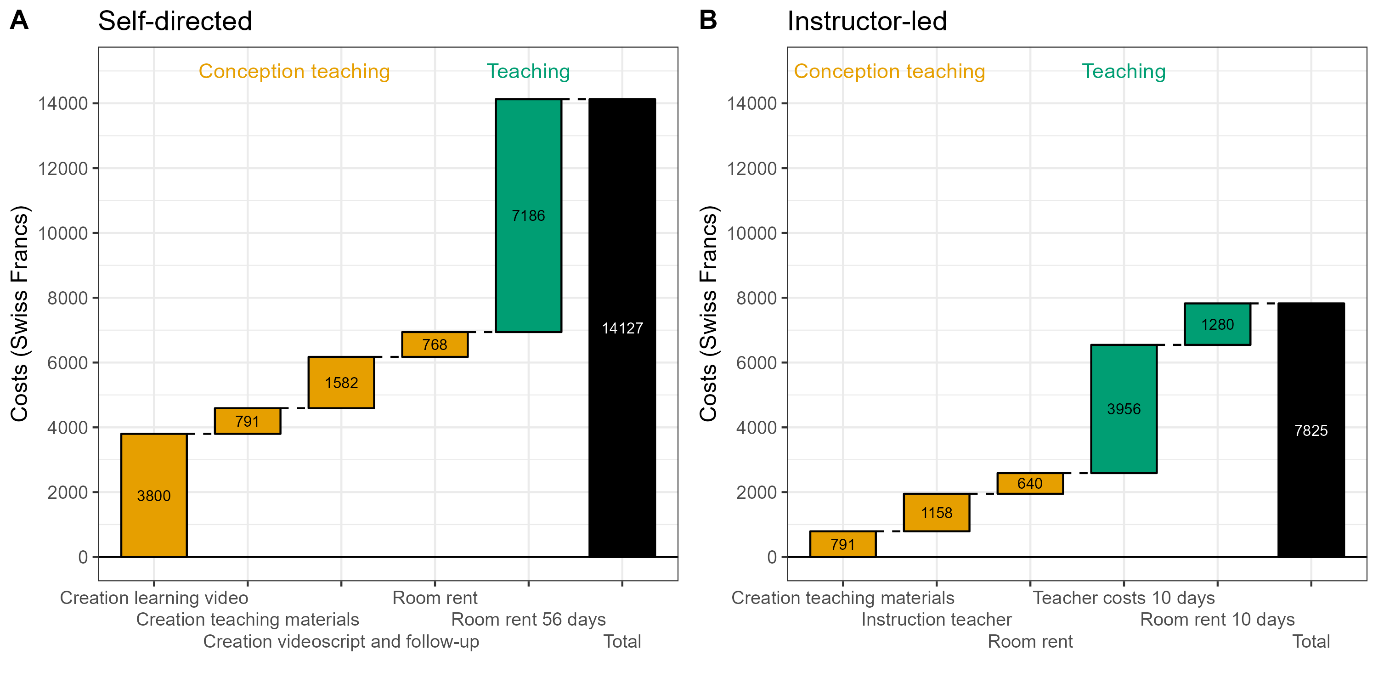
**

**Supplementary Figure 2.** Waterfall plot of the cost calculation of training creation and training for (A) self-directed and (B) instructor led teaching.
